# Underwater hybrid ESD versus conventional ESD for colorectal laterally spreading tumors: a retrospective study

**DOI:** 10.3389/fmed.2026.1942087

**Published:** 2026-10-01

**Authors:** Qirun Cheng, Yu-liang Feng, Li-peng Chen

**Affiliations:** Department of Gastroenterology, Zhejiang Hospital, Hangzhou, Zhejiang, China

**Keywords:** colorectal neoplasms, hybrid endoscopic submucosal dissection, laterally spreading tumors, R0 resection, underwater endoscopic submucosal dissection

## Abstract

**Background and aims:**

Conventional endoscopic submucosal dissection (C-ESD) is standard for colorectal laterally spreading tumors (LSTs) but is time-consuming. Underwater hybrid ESD (U-Hybrid ESD) is an emerging technique. We compared the efficacy and safety of U-Hybrid ESD versus C-ESD for colorectal LSTs.

**Methods:**

This exploratory, single-center retrospective study included 300 patients with LSTs ≥20 mm (U-Hybrid ESD, *n* = 150; C-ESD, *n* = 150). Procedure time, postoperative hospital stay, time to resume oral diet, R0 resection rate, and adverse events were compared. Hypothesis-generating stratified analyses by lesion size and pathology were also performed.

**Results:**

After multivariable adjustment, U-Hybrid ESD had significantly shorter procedure time (adjusted mean 21.29 vs. 31.99 min, adjusted mean difference −10.70, *P* < 0.001), shorter hospital stay (2.38 vs. 2.61 days, *P* = 0.008), and earlier oral diet (25.28 vs. 28.32 h, *P* = 0.004). The R0 resection rate showed a numerical difference between groups (96.67% vs. 100%), which did not reach statistical significance on Fisher's exact test (*P* = 0.060). Adverse events did not differ significantly (5.33% vs. 9.33%, *P* = 0.318).

**Conclusions:**

In this exploratory analysis, U-Hybrid ESD was associated with shorter procedure time and modest improvements in postoperative recovery metrics, without a significant increase in complications. These hypothesis-generating findings suggest that U-Hybrid ESD may offer efficiency benefits for selected lesions, but the potential trend toward lower R0 rates for large or high-grade lesions warrants caution and prospective validation before any definitive size-based recommendations can be made.

## Introduction

Colorectal laterally spreading tumors (LSTs) are non-polypoid lesions ≥10 mm in diameter that extend circumferentially along the colonic wall rather than vertically (1). They have significant malignant potential, with the risk of submucosal invasion increasing with lesion size and the presence of nodular or depressed areas (2, 3). Endoscopic submucosal dissection (ESD) enables high *en bloc* and R0 resection rates and is therefore the preferred treatment for large (≥20 mm) LSTs (4, 5).

Nevertheless, conventional ESD (C-ESD) is technically challenging, has a long learning curve, and is associated with prolonged procedure time and non-negligible risks of perforation and bleeding (6, 7). Various modifications have been developed to simplify the procedure. Hybrid ESD combines partial submucosal dissection with snare resection, reducing time and difficulty (8, 9). Underwater EMR (UEMR) uses water instead of air, utilizing buoyancy to separate mucosal and muscular layers, often without submucosal injection (10, 11). UEMR is effective for intermediate-sized polyps but yields lower *en bloc* resection rates for large LSTs (12).

Underwater hybrid ESD (U-Hybrid ESD) integrates these principles: water immersion for better visualization, submucosal injection, partial circumferential incision and limited dissection, followed by snare resection. This technique has been described in small case series (13–15) but has never been directly compared with C-ESD for LSTs in a relatively large cohort. Therefore, this retrospective study aimed to compare the perioperative outcomes of U-Hybrid ESD versus C-ESD for colorectal LSTs and to identify optimal indications for each technique.

## Methods

### Study design and patients

This single-center retrospective study included consecutive patients who underwent endoscopic resection for colorectal LSTs (diameter ≥20 mm) between April 2023 and December 2025 at Zhejiang Hospital (Hangzhou, China). Inclusion criteria: (1) endoscopic diagnosis of LST (granular or non-granular); (2) lesion size ≥20 mm; (3) treatment by U-Hybrid ESD or C-ESD; (4) complete clinical and pathological data. Exclusion criteria: (1) deep submucosal invasion (≥T1b) or lymph node/distant metastasis confirmed by endoscopic ultrasonography or computed tomography; (2) prior endoscopic or surgical treatment for the same lesion; (3) coagulopathy or failure to discontinue antiplatelet/anticoagulation therapy; (4) overt invasive cancer on pre-procedural biopsy. A total of 300 patients (150 in each group) were enrolled. Treatment allocation (U-Hybrid ESD vs. C-ESD) was at the discretion of the attending endoscopists based on their clinical judgment and preference. No patients were excluded to balance group sizes; the equal numbers in each group occurred coincidentally after applying all inclusion and exclusion criteria.

U-Hybrid ESD was introduced at our institution in April 2023. After its introduction, both U-Hybrid ESD and conventional C-ESD were performed contemporaneously. The selection of resection technique was at the discretion of the performing endoscopist, based on lesion size, morphology, pre-procedural optical assessment, and operator experience, without randomization. Procedures were performed by four experienced endoscopists. The case distribution between the two techniques was as follows: Operator A: U-Hybrid ESD [38]/C-ESD [42]; Operator B: U-Hybrid ESD [35]/C-ESD [40]; Operator C: U-Hybrid ESD [40]/C-ESD [35]; Operator D: U-Hybrid ESD [37]/C-ESD [33].

### Ethical approval

The study was conducted in accordance with the Declaration of Helsinki. The protocol was approved by the Medical Ethics Committee of Zhejiang Hospital (Approval No. 2020-60 K). Written informed consent was waived due to the retrospective nature of the study. Patient data were anonymized prior to analysis.

### Procedures

All procedures were performed by four experienced endoscopists, each with >3 years of colorectal ESD experience and having performed >100 colorectal ESD cases independently. High-definition colonoscopes (PCF-Q260J or CF-H290, Olympus, Tokyo, Japan) with a transparent hood were used. An electrosurgical unit (VIO300D, ERBE, Tübingen, Germany) was employed. A mucosal incision knife manufactured by Vedkang (Jiangsu Vedkang Medical Science and Technology Co., Ltd., China) was used for both techniques. Submucosal injection was performed with 2–5 mL of solution per point. All patients discontinued antiplatelet/anticoagulation drugs for 5–7 days before the procedure without bridging therapy. En bloc resection was attempted for all lesions in both groups.

C-ESD group: After marking the periphery (2–3 mm outside the lesion), submucosal injection of normal saline mixed with methylene blue and diluted epinephrine (1:100,000) was performed to lift the lesion. A circumferential incision was made using the Vedkang knife, followed by stepwise submucosal dissection until complete *en bloc* resection. Visible vessels were coagulated with hemostatic forceps (FD-410LR, Olympus). Prophylactic clipping was performed at the endoscopist's discretion.

U-Hybrid ESD group: After locating the lesion, intraluminal air was aspirated and warm normal saline (37 °C) was infused to completely immerse the lesion. The lesion was examined under water to identify margins. Submucosal injection (same solution: normal saline + methylene blue + diluted epinephrine 1:100,000) was then performed to elevate the lesion. A partial circumferential incision was made with the Vedkang knife, and limited submucosal dissection was carried out to expose the base of the lesion (approximately 50%–70% of the lesion circumference). Subsequently, an appropriately sized snare (JiuHong Medical, Changzhou, China) was used to capture the remaining undissected portion, and resection was completed using blended current (EndoCut Q, effect 3). Post-resection management (hemostasis, clipping) was identical to C-ESD.

### Definitions and data collection

Procedure time: from the start of lesion marking to the completion of resection, hemostasis and clip closure (minutes), consistent for both groups. Postoperative hospital stay: days from procedure to discharge. Time to resume oral diet: hours from procedure to first oral intake. All pathological evaluations were performed by two experienced pathologists who were blinded to the treatment group. R0 resection was defined as tumor-free lateral and vertical margins ≥1 mm on histology (4). R1 indicated margin positivity. Adverse events: delayed bleeding (hematochezia requiring endoscopic hemostasis within 72 h (16), electrocoagulation syndrome (post-procedural abdominal pain, fever, leukocytosis without perforation). Perforation was recorded if a visible hole or free air on imaging.

### Statistical analysis

SPSS version 23.0 (IBM Corp., Armonk, NY, USA) was used. Continuous variables were expressed as mea*n* ± standard deviation (SD) or median (interquartile range) as appropriate. The normality was tested using the Shapiro–Wilk test. Normally distributed data were compared using the independent t-test; non-normally distributed data were analyzed using the Mann–Whitney U test. Categorical variables are presented as *n* (%) and were compared by *χ*^2^ test or Fisher's exact test. Multivariable linear regression analysis was performed to adjust for potential confounders (age, sex, lesion location, morphology, size, pathology, and treating endoscopist) when comparing procedure time between the two groups. Because the C-ESD group had zero events for R0 resection failure, multivariable logistic regression was not feasible for R0 comparison; instead, stratified analyses by pathological type and lesion size were conducted. A two-sided *P* < 0.05 was considered statistically significant. To formally evaluate whether lesion size modified the treatment effect on R0 resection, we added a multiplicative interaction term (treatment group  ×  lesion size as a continuous variable) to the multivariable logistic regression model. Owing to the sparse events (only 5 R0 failures), we used Firth's penalized likelihood approach for this analysis. A two-sided *P* < 0.05 was considered statistically significant for the interaction term.

The selection process is detailed in Figure 1.

**Figure 1:**
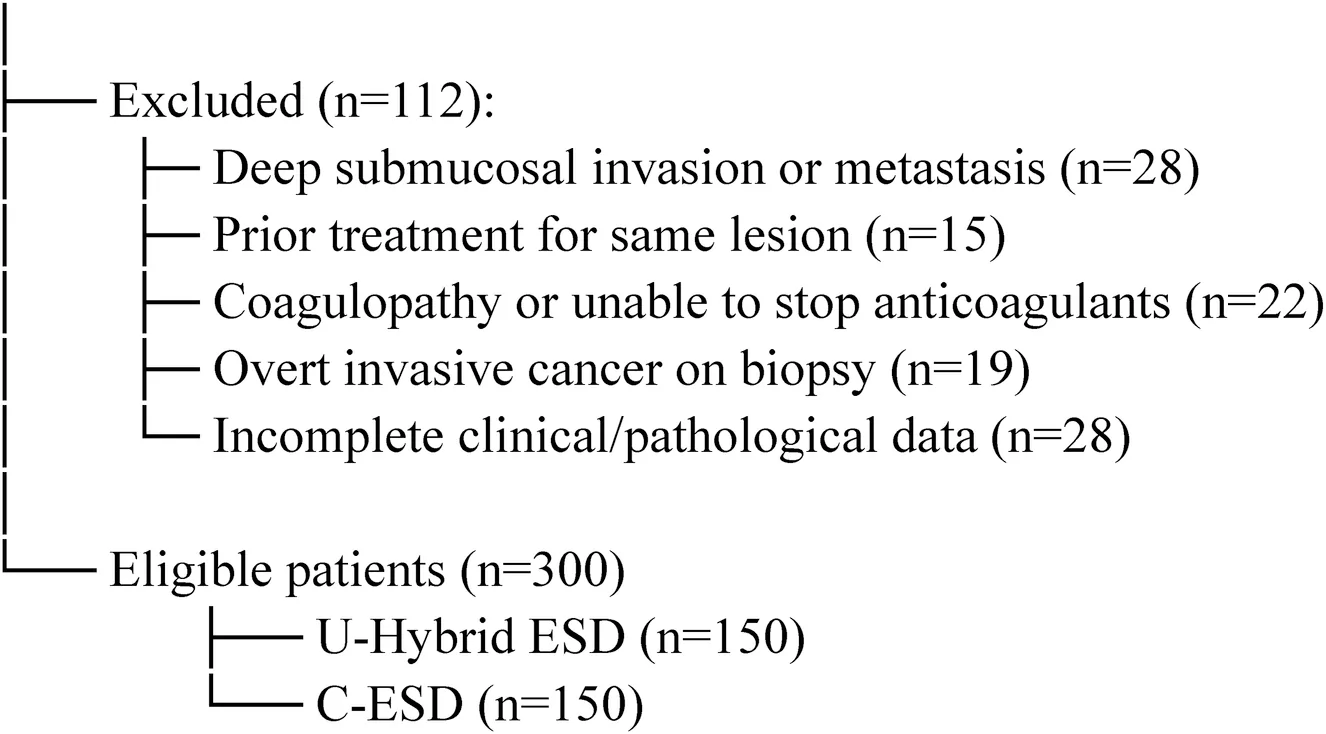
Patient selection flowchart. Patients who underwent endoscopic resection for colorectal LSTs (≥20 mm) between Apr 2023 and Dec 2025 (*n* = 412).

## Results

### Baseline characteristics

All 300 patients were included in the final analysis with no dropouts. Baseline characteristics are shown in Table 1. No significant differences were found in age, sex, or lesion location. However, the U-Hybrid ESD group had a smaller mean lesion size (29.77 ± 5.73 mm vs. 31.68 ± 6.35 mm, *P* = 0.007) and a lower proportion of non-granular lesions (43.33% vs. 55.33%, *P* = 0.038). Pathological types were comparable between groups (*P* = 0.181).

**Table 1:** Baseline characteristics of patients.

Characteristic	U-Hybrid ESD (*n* = 150)	C-ESD (*n* = 150)	*P*-value
Age (years), mean ± SD	51.55 ± 9.85	53.21 ± 9.39	0.136
Male sex, *n* (%)	73 (48.67)	65 (43.33)	0.374
Lesion location (right colon), *n* (%)	91 (60.67)	86 (57.33)	0.557
Non-granular morphology, *n* (%)	65 (43.33)	83 (55.33)	0.038
Lesion size (mm), mean ± SD	29.77 ± 5.73	31.68 ± 6.35	0.007
Pathologic type, *n* (%)			0.181
Adenoma	130 (86.67)	118 (78.67)	
HGIN	15 (10.00)	25 (16.67)	
Intramucosal carcinoma	5 (3.33)	7 (4.67)	

HGIN, high-grade intraepithelial neoplasia. Data are presented as mea*n* ± SD or *n* (%). *P* values were calculated using the independent t-test for age and lesion size, and chi-square test for sex, location, morphology, and pathology. The C-ESD group had significantly larger lesion size and more non-granular lesions, reflecting real-world allocation bias.

### Overall perioperative outcomes

After multivariable adjustment, U-Hybrid ESD was associated with significantly shorter procedure time (adjusted mean 21.29 vs. 31.99 min, adjusted MD −10.70, 95% CI −12.56 to −8.84, *P* < 0.001), shorter hospital stay (adjusted mean 2.38 vs. 2.61 days, MD −0.22, 95% CI −0.38 to −0.06, *P* = 0.008), and earlier oral diet resumption (adjusted mean 25.28 vs. 28.32 h, MD −2.98, 95% CI −5.01 to −0.95, *P* = 0.004). En bloc resection was achieved in all lesions in both the U-Hybrid ESD and C-ESD groups (100%, 150/150 for each group). The R0 resection rate showed a numerical difference (96.67% vs. 100%), which did not reach statistical significance (Fisher's exact *P* = 0.060). Adverse events were not significantly different (5.33% vs. 9.33%; adjusted OR 0.50, 95% CI 0.20–1.25, *P* = 0.138). Detailed adverse events are shown in Table 2. The interaction between treatment group and lesion size (as a continuous variable) was not statistically significant for R0 resection (*P* for interactio*n* = 0.51). No perforations occurred in either group.

**Table 2:** Overall comparison of perioperative outcomes.

Outcome	U-Hybrid ESD(*n* = 150)	C-ESD(*n* = 150)	Crude difference/OR (95% CI)	Adjusted difference/OR* (95% CI)	*P*-value
Procedure time (min), mean ± SD	21.41 ± 4.34	29.70 ± 4.47	MD −8.29 (−9.30, −7.28)	MD −10.70 (−12.56, −8.84)	<0.001
Postoperative hospital stay (days), mean ± SD	2.38 ± 0.70	2.61 ± 0.77	MD −0.23 (−0.40, −0.06)	MD −0.22 (−0.38, −0.06)	0.008
Time to resume oral diet (h), mean ± SD	25.28 ± 8.47	28.32 ± 9.76	MD −3.04 (−5.13, −0.95)	MD −2.98 (−5.01, −0.95)	0.004
R0 resection, *n* (%)	145 (96.67)	150 (100.00)	Not applicable^a^	Not applicable^a^	0.060^b^
Adverse events, *n* (%)	8 (5.33)	14 (9.33)	OR 0.55 (0.22, 1.36)	OR 0.50 (0.20, 1.25)^c^	0.138^c^
Detailed adverse events					
Delayed bleeding, *n* (%)	6 (4.00)	8 (5.33)			
Electrocoagulation syndrome, *n* (%)	2 (1.33)	6 (4.00)			
Perforation, *n* (%)	0 (0)	0 (0)			

Adjusted for age, sex, lesion location, morphology, size, and pathology using multivariable linear regression (for continuous outcomes) or Firth penalized logistic regression (for adverse events). ^a^R0 failure occurred only in the U-Hybrid group (5 events); multivariable regression was not feasible. ^b^P value from Fisher's exact test. ^c^Adjusted OR and *P* from Firth penalized regression; interpretation should be cautious due to sparse events.

The symbol * indicates adjusted estimates, adjusted for age, sex, lesion location, morphology, size, and pathology.

### Stratified analysis by pathological type with adjustment for procedure time

After adjustment using multivariable linear regression, procedure time remained significantly shorter for U-Hybrid ESD (adjusted mea*n* ± SE: 21.29 ± 0.76 vs. 31.99 ± 0.67 min, adjusted mean difference −10.70, 95% CI −12.56 to −8.84, *P* < 0.001). For adenomas, U-Hybrid ESD had comparable R0 resection (97.69% vs. 100%, *P* = 0.249) but significantly shorter hospital stay (2.25 ± 0.63 vs. 2.53 ± 0.65 days, *P* = 0.002) and earlier oral intake (24.74 ± 8.15 vs. 27.97 ± 9.80 h, *P* = 0.001). For HGIN and IMC, R0 rates were lower in the U-Hybrid ESD group (93.33% and 80.00%, respectively) but differences were not statistically significant. Stratified by final pathology, the R0 rates for adenoma, HGIN, and IMC in the U-Hybrid group were 97.7% (127/130), 93.3% (14/15), and 80.0% (4/5), respectively, compared with 100% in all C-ESD subgroups. These differences were not statistically significant (Fisher's exact *P* = 0.249, 0.375, and 0.417, respectively; full data in Supplementary Table S1)

### Stratified analysis by lesion size

Stratified by lesion size (Table 3), U-Hybrid ESD significantly reduced procedure time in all subgroups (all *P* < 0.001). For lesions <30 mm and 30–39 mm, R0 and adverse event rates were similar between groups. In the exploratory ≥40 mm subgroup (*n* = 6 in U-Hybrid group), R0 resection was 66.7% vs. 100% (*P* = 0.079, Fisher's exact test).

**Table 3:** Stratified analysis by lesion size.

Lesion size/Outcome	U-Hybrid ESD	C-ESD	*P*-value
<30 mm	*N* = 86	*N* = 68	-
Procedure time (min)	21.86 ± 4.63	28.32 ± 3.29	<0.001
Postop hospital stay (days)	2.30 ± 0.62	2.49 ± 0.66	0.071
Time to oral diet (h)	24.8 ± 8.2	28.1 ± 9.5	0.023
R0 resection	84/86 (97.67)	68/68 (100.00)	0.504
Adverse events	1/86 (1.16)	1/68 (1.47)	1.000
30–39 mm	*N* = 58	*N* = 68	-
Procedure time (min)	20.90 ± 3.87	30.32 ± 4.64	<0.001
Postop hospital stay (days)	2.45 ± 0.78	2.72 ± 0.85	0.067
Time to oral diet (h)	25.6 ± 8.6	28.6 ± 10.0	0.080
R0 resection	57/58 (98.28)	68/68 (100.00)	0.460
Adverse events	5/58 (8.62)	7/68 (10.29)	0.411
≥40 mm	*N* = 6	*N* = 14	-
Procedure time (min)	19.83 ± 4.22	33.36 ± 6.02	<0.001
Postop hospital stay (days)	3.67 ± 1.03	4.07 ± 1.49	0.551
Time to oral diet (h)	36.0 ± 17.9	38.6 ± 13.4	0.729
R0 resection	4/6 (66.67)	14/14 (100.00)	0.079^a^
Adverse events	2/6 (33.33)	6/14 (42.86)	1.000^a^

a Fisher's exact test. All subgroup analyses are exploratory and were not adjusted for multiple comparisons. The ≥40 mm subgroup contains only 6 cases in the U-Hybrid group; the 95% confidence interval for the R0 rate is 22.3%–95.7%, indicating substantial uncertainty. These results should be interpreted as hypothesis-generating.

## Discussion

Given the retrospective and exploratory nature of this study, all subgroup analyses were considered hypothesis-generating. We did not adjust for multiple comparisons, and thus these findings should be interpreted cautiously and require confirmation in future prospective studies. This retrospective study directly compared U-Hybrid ESD and C-ESD for colorectal LSTs and demonstrated that U-Hybrid ESD offers significantly shorter procedure time and modest improvements in postoperative recovery metrics without an increase in adverse events. However, the numerical trend toward a lower R0 resection rate with U-Hybrid ESD, especially for lesions ≥40 mm and those with high-grade histology, suggests that careful patient selection may be important, but given the exploratory nature of our subgroup analyses, this hypothesis requires prospective confirmation. Nevertheless, due to the retrospective design, these findings should be considered exploratory rather than confirmatory.

### Procedure time and recovery advantages

The reduction in procedure time (approximately 8–10 min on average) is clinically meaningful. Prolonged ESD is associated with higher sedation requirements, increased patient discomfort, greater operator fatigue, and potentially high costs (17). U-Hybrid ESD shortens the procedure by limiting the extent of submucosal dissection; after partial circumferential incision and limited dissection, the remaining tissue is removed by snaring. This approach is less demanding and faster while still providing *en bloc* resection in most cases. Similar findings have been reported for hybrid ESD in colorectal and gastric lesions (8, 9, 18).

The underwater environment likely contributes to the time saving. Water immersion reduces light reflection, magnifies mucosal structures, and helps identify lesion margins (10). The buoyancy of the lesion facilitates snaring. Moreover, the heat-sink effect of water reduces thermal injury and may decrease the need for extensive hemostasis (19). Recent studies have further demonstrated that the underwater technique can significantly improve dissection speed compared with conventional ESD, likely due to the sustained tissue tension and clearer visualization (20, 21). Faster recovery (shorter hospital stay and earlier oral diet) reflects reduced tissue trauma and a lower incidence of post-procedural pain and ileus. Although the approximately 3-hour earlier oral intake was statistically significant, its clinical relevance is uncertain and may depend on institutional protocols.

### R0 resection rate: the trade-off for efficiency

The most critical observation was the numerical trend toward a lower R0 resection rate in the U-Hybrid ESD group (96.67% vs. 100%), although this difference did not reach statistical significance (*P* = 0.060). Nevertheless, R0 resection is the goal of curative endoscopic treatment because positive margins are associated with higher local recurrence (22, 23). The numerical difference was primarily observed in the exploratory ≥40 mm subgroup, where U-Hybrid ESD achieved 66.67% R0 resection (4/6) compared with 100% in the C-ESD group. However, this subgroup included only 6 U-Hybrid cases, and the difference was not statistically significant (*P* = 0.079). Therefore, this observation should be considered hypothesis-generating and requires prospective validation before any definitive conclusions can be drawn. In contrast, C-ESD provides direct visual control of the dissection plane, enabling complete removal even for giant or fibrotic lesions (4, 24).

Our stratified analysis by pathology showed that for pure adenomas, U-Hybrid ESD had a high R0 rate (97.69%) that was not significantly different from C-ESD (100%). However, for HGIN and IMC, U-Hybrid ESD had lower R0 rates (93.33% and 80.00%, respectively). Although these differences were not statistically significant (likely due to small sample sizes, type II error), this numerical trend raises concern. For lesions with high-grade dysplasia or early cancer, even a small risk of incomplete resection is unacceptable because it may lead to disease progression or require additional surgery (25). Although we observed numerically lower R0 rates in lesions with HGIN/IMC, these were post-resection diagnoses and thus cannot inform pre-procedural decision-making. Future studies should evaluate whether pre-operative optical features (e.g., pit pattern, vascular pattern, morphology) can identify lesions at risk for incomplete hybrid resection.

Our results align with previous studies. A meta-analysis by Ni et al. (26) showed that underwater EMR had a higher *en bloc* resection rate than conventional EMR for polyps ≥20 mm. However, that study did not include ESD. Hybrid ESD trials by Bae et al. (8) reported similar R0 rates between hybrid and conventional ESD for colorectal tumors (mean size 22 mm). Our study extends these findings to larger LSTs and highlights that for lesions ≥40 mm or with high-grade histology, the hybrid approach may be inadequate.

Recently, a large international multicenter study of 547 colonic ESDs [Karna et al. (27)] reported that while most colon ESDs are performed for benign pathology, non-curative resection rates remain high for lesions containing cancer (26.5% for T1 cancers), underscoring the critical importance of appropriate lesion selection. This aligns with our hypothesis that U-Hybrid ESD may be more suitable for low-risk lesions, but this requires prospective validation.

It is important to note that the stratified analyses by final pathology were *post-hoc* and cannot inform pre-procedural decision-making, since pathological diagnosis is only available after resection. Future studies should evaluate whether pre-procedural optical features can identify lesions at risk for incomplete hybrid resection. Due to the very small sample size, this finding should be interpreted with caution and does not establish a definitive size threshold.

### Safety profile

Adverse events were low in both groups and not significantly different. The numerically lower rate of electrocoagulation syndrome in the U-Hybrid ESD group (1.33% vs. 4.00%) may be explained by the protective effect of water, which dissipates heat and reduces transmural thermal spread (10, 22). No perforations occurred in either group, reflecting both the high level of operator experience (>100 colorectal ESD cases each) and the safety of the hybrid technique when applied to appropriate lesions. Delayed bleeding rates (4.00% vs. 5.33%) were comparable to those reported in large ESD series (16, 27). The favorable safety profile of underwater techniques is further supported by a recent propensity score-matched analysis by Andrisani et al. (28), which compared underwater ESD with conventional ESD for superficial colorectal neoplasms. They found a significantly lower perforation rate in the underwater group (1% vs. 9.5%, *P* = 0.02) while maintaining a 100% *en bloc* resection rate, reinforcing the potential safety benefits of underwater dissection.

### Clinical implications

Based on these hypothesis-generating findings, we hypothesize that U-Hybrid ESD may offer efficiency benefits for smaller lesions, but the present data do not support a definitive size threshold. We suggest that U-Hybrid ESD may be more suitable for low-risk lesions until prospective trials define optimal indications. However, given the *post-hoc* nature of the subgroup analyses and the small sample sizes, these hypotheses require validation in well-designed prospective studies before any firm clinical recommendations can be made.

### Comparison with prior literature

The concept of underwater hybrid ESD was previously described in case reports and small series [Rodríguez-Sánchez et al., 2018; Figueiredo et al., 2022; Gaulin et al., 2026] (13–15). Our study is the first, to our knowledge, to directly compare this technique with C-ESD for colorectal LSTs in a relatively large cohort. A comprehensive technical review by the European Society of Gastrointestinal Endoscopy [Libânio et al. (29)] has summarized the evolving landscape of ESD techniques, including hybrid and underwater methods. To our knowledge, this is the largest single-center study that directly compares U-Hybrid ESD and C-ESD specifically for colorectal LSTs. Previous studies have compared UEMR with EMR (12) or hybrid ESD with conventional ESD for mixed colorectal neoplasms (8, 9). Our study provides new insights by focusing on LSTs and using detailed stratified analyses by size and pathology. The suggestive trend that U-Hybrid ESD may be comparable for lesions <30 mm but potentially less effective for lesions ≥40 mm provides a hypothesis that requires prospective validation. Recent RCTs have also suggested potential advantages of underwater ESD for selected large LSTs (30), supporting the plausibility of our observations.

### Limitations

Several limitations should be acknowledged. First, the retrospective design and non-randomized allocation may have introduced selection bias. Baseline imbalances in lesion size and morphology suggest that endoscopists preferentially assigned more challenging lesions to C-ESD. Although we adjusted for measured confounders, residual confounding (e.g., submucosal fibrosis, intraoperative difficulty) cannot be excluded. Recent evidence (31) has highlighted that fibrosis is a key determinant of ESD success and may influence the choice of hybrid versus conventional approach. Our study did not collect fibrosis data, which is a notable limitation. A recent large multicenter retrospective study demonstrated that both conventional ESD and hybrid ESD can achieve effective endoscopic treatment for fibrotic, non-lifting colorectal lesions, and importantly identified the degree of fibrosis as one of the key factors associated with successful completion of conventional ESD without conversion to a hybrid approach (31). Furthermore, for selected technically challenging lesions where conventional dissection or snare capture may be problematic, advanced techniques such as dual-channel, double-traction device-assisted endoscopic full-thickness resection have been described as complementary therapeutic options (32). These reports underscore that management of fibrotic or non-lifting colorectal lesions requires individualized decision-making, and the absence of fibrosis assessment in our retrospective cohort may have influenced the observed outcomes. Second, the lack of randomization precludes causal inference. Third, the sample size in the ≥40 mm subgroup (*n* = 6 in U-Hybrid ESD) was small; larger studies are needed to confirm the size threshold. The wide 95% confidence interval for the R0 rate in this subgroup (22.3% to 95.7%) reflects this imprecision. Fourth, long-term recurrence data were not available. Although R0 resection is a surrogate for cure, actual recurrence after R1 resection may be lower due to cautery effects (33). Future studies with long-term surveillance are needed to confirm the oncological outcomes of U-Hybrid ESD. Fifth, this was a single-center study, and generalizability to other settings may be limited.

Despite these limitations, this study has strengths: it is the first direct comparison for LSTs; the stratified analyses provide actionable guidance; the homogenous cohort (LSTs ≥20 mm) and standardized definitions enhance internal validity; and the use of a common knife brand (Vedkang) and injection solution (saline + methylene blue) reflects real-world practice.

### Future directions

Prospective randomized controlled trials are needed to confirm our findings and to establish optimal size and histology thresholds for U-Hybrid ESD. Cost-effectiveness analyses comparing the two techniques (including procedure time, consumables, hospital stay, and recurrence management) would also be valuable. Technological improvements, such as dedicated underwater snares or more viscous injection solutions, might further improve R0 resection rates.

## Conclusions

In this exploratory study, U-Hybrid ESD was associated with shorter procedure time and modest improvements in postoperative recovery metrics compared with C-ESD for colorectal LSTs, without increasing complications. However, the numerical trend toward a lower R0 resection rate in exploratory subgroup analyses, particularly in larger lesions, suggests caution in applying this technique to high-risk lesions without prospective validation. Our findings are hypothesis-generating, and prospective randomized trials are warranted to validate these observations and to define optimal patient selection.

## Data Availability

The raw data supporting the conclusions of this article will be made available by the authors, without undue reservation.
