# Supplementary material for: Underwater hybrid ESD versus conventional ESD for colorectal laterally spreading tumors: a retrospective study
Source: Front Med (Lausanne). 2026 Oct 1;13:1942087. doi: 10.3389/fmed.2026.1942087 (PMC13668835; doi:10.3389/fmed.2026.1942087)
Supplement: Supplementary file 1 [file Table1.docx]

Supplementary Table S1. Stratified outcomes by pathological type

| Outcome / Pathologic type | U-Hybrid ESD | C-ESD | OR / mean difference (95% CI) | P-value |
| --- | --- | --- | --- | --- |
| R0 resection, n (%) | | | | |
| Adenoma | 127/130 (97.69) | 118/118 (100.00) | Not calculated | 0.249 |
| HGIN | 14/15 (93.33) | 25/25 (100.00) | Not calculated | 0.375 |
| IMC | 4/5 (80.00) | 7/7 (100.00) | Not calculated | 0.417 |
| Adverse events, n (%) | | | | |
| Adenoma | 2/130 (1.54) | 6/118 (6.08) | Not calculated | 0.269 |
| HGIN | 4/15 (26.67) | 7/25 (28.00) | Not calculated | 0.311 |
| IMC | 2/5 (40.00) | 1/7 (14.29) | Not calculated | 0.424 |
| Postop hospital stay (days), mean ± SD | | | | |
| Adenoma | 2.25±0.63 | 2.53±0.65 | - | 0.002 |
| HGIN | 3.07±0.46 | 2.96±1.06 | - | 0.376 |
| IMC | 3.60±0.89 | 2.71±1.11 | - | 0.106 |
| Time to oral diet (h), mean ± SD | | | | |
| Adenoma | 24.74±8.15 | 27.97±9.80 | - | 0.001 |
| HGIN | 28.80±9.94 | 30.24±9.87 | - | 0.619 |
| IMC | 28.80±10.73 | 27.43±9.07 | - | 0.876 |
| Procedure time (min) | | | | |
| Overall | 21.41±4.34 | 29.70±4.47 | - | <0.001† |

HGIN, high‑grade intraepithelial neoplasia; IMC, intramucosal carcinoma; MD, mean difference; CI, confidence interval. †The shorter procedure time in the U-Hybrid ESD group remained significant after multivariable adjustment (adjusted mean difference -10.70 min, 95% CI -12.56 to -8.84, P<0.001).
